# Scimitar Syndrome: Late Presentation and Conservative Management

**DOI:** 10.7759/cureus.1997

**Published:** 2017-12-28

**Authors:** Mohanad Saleh, Murad Abdelsalam, Anupam A Sule, Michele Degregorio

**Affiliations:** 1 Department of Internal Medicine, St. Joseph Mercy Oakland, Pontiac, USA; 2 Department of Cardiology, St. Joseph Mercy Oakland, Pontiac, USA

**Keywords:** partial anomalous pulmonary venous return, scimitar syndrome, pulmonary hypertension, conservative treatment, congenital anomaly

## Abstract

Partial anomalous pulmonary venous return (PAPVR) is a rare congenital malformation. The infracardiac variant with the right lobe of the lung draining to the inferior vena cava (IVC) is called Scimitar syndrome. The infantile subtype presents before one year of age and the adult variant is also usually diagnosed in childhood.

A 70-year-old woman presented with worsening shortness of breath. An echocardiogram suggested severe pulmonary hypertension that was confirmed by right heart catheterization. A computed tomography (CT) without contrast revealed an anomalous vein from the right upper lobe suggestive of Scimitar syndrome. The patient did not have any other associated congenital heart defects (CHD) (incomplete Scimitar syndrome). A surgical treatment approach was avoided due to the incomplete nature of the Scimitar syndrome.

Incomplete Scimitar syndrome may present later and with less severity than the typical Scimitar syndrome with left to right shunting occurring only in the lung and may be managed nonsurgically.

## Introduction

Infracardiac partial anomalous pulmonary venous return (PAPVR) is a rare congenital venous anomaly (one in a million live births) in which the right pulmonary veins drain to the inferior vena cava (IVC) [1]. This anomalous vein appears similar to a Turkish curved sword (the eponymous Scimitar) on a chest x-ray. The infantile variant presents before one year of age. The adult variant presents later and a median age of presentation has not been reported. Diagnosis of this anomaly and determining the management strategy can be challenging especially in patients who present at an older age.

## Case presentation

Presentation

A 71-year-old lady had originally presented in 2015 with chest pain radiating to the back. An echocardiogram performed at that admission had noted elevated right ventricular systolic pressure and she had been advised to follow up for further evaluation. She presented in 2017 with dyspnea on exertion for two weeks. She did not have any chest pain, fever, diaphoresis, cough, wheezing, orthopnea, palpitations, syncope or presyncope.

Past medical history included hypertension, hyperlipidemia, diabetes mellitus and chronic kidney disease stage III. She did not have a history of recurrent respiratory infections.

She had worked in the car industry with some exposure to chemical fumes. The patient denied ever having smoked or used recreational drugs. Her home medications included pantoprazole, clopidogrel, simvastatin, levothyroxine, venlafaxine, and glyburide.

The patient’s blood pressure was elevated at 153/103. The other vital signs were within normal limits. She had a regular rhythm and pulse at a normal rate. Jugular venous distension was absent. No murmurs or bruits were heard but the patient had a loud P2 over the pulmonic area. Leg edema was absent. Auscultation of the lungs revealed coarse breath sounds over the right lower lobe but the rest of the lung fields were normal without any crackles or wheezes.

Investigations

**Figure 1:**
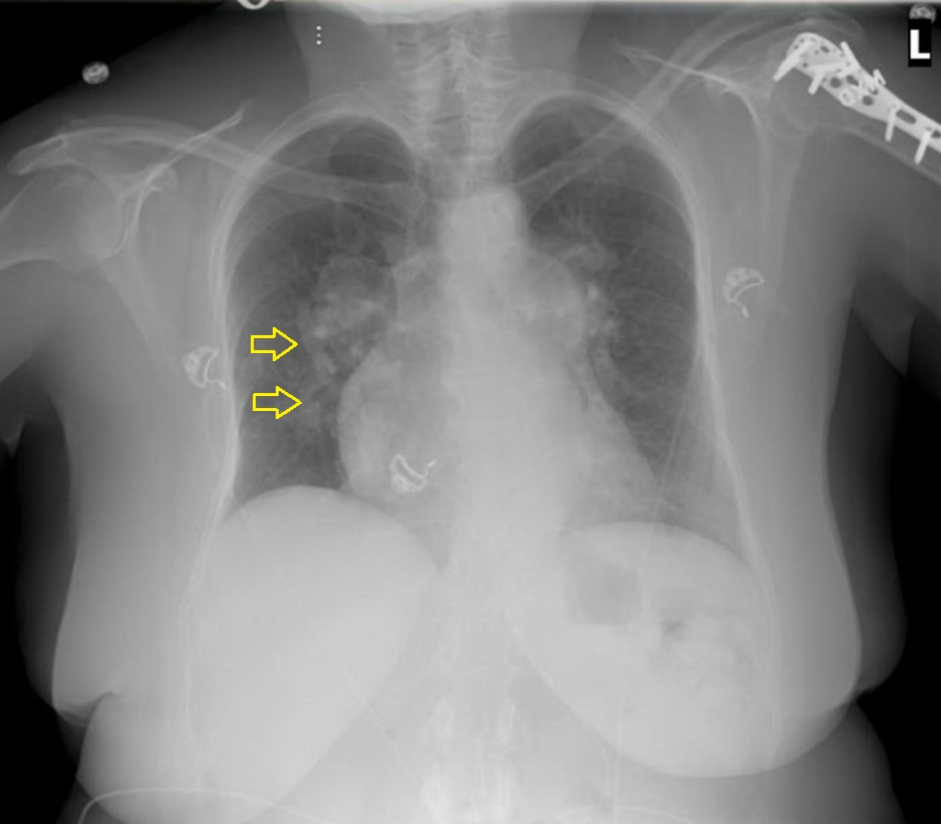
Chest X-ray demonstrating the Scimitar vein (marked by yellow arrow).

A chest X-ray revealed stable cardiomegaly with prominence of the bilateral hila consistent with enlarged pulmonary arteries. A Scimitar vein was also seen (Figure 1).

**Figure 2:**
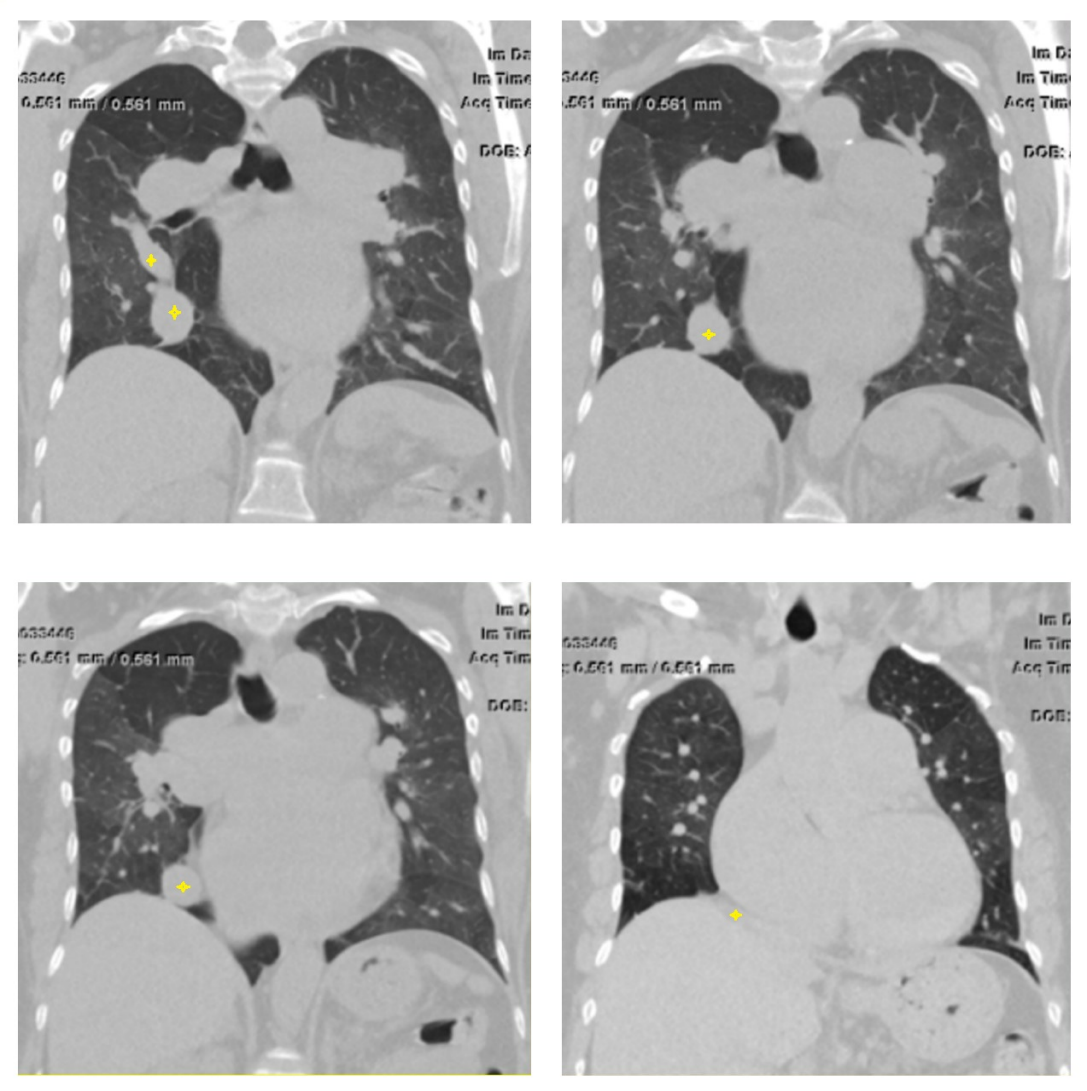
Computed tomography scan of chest (coronal reconstruction) demonstrating the Scimitar vein (marked by the yellow cross).

**Figure 3:**
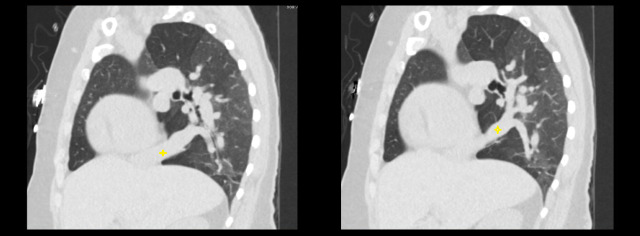
Computed tomography scan of chest (sagittal reconstruction) demonstrating the Scimitar vein (marked by the yellow cross).

A computed tomography (CT) scan of her chest without contrast identified mosaic interstitial lung disease and the presence of an anomalous vessel in the right lower lobe which was draining into the IVC (Figures 2, 3).

Magnetic resonance angiography confirmed the presence of isolated infracardiac PAPVR (incomplete Scimitar syndrome).

An echocardiogram revealed preserved ejection fraction and grade II diastolic dysfunction. A moderately dilated left atrium was seen without a patent foramen ovale on bubble study. Right atrial enlargement, severe tricuspid regurgitation and right ventricular systolic pressure of 91 mmHg indicated a high likelihood of severe pulmonary hypertension.

Right heart catheterization confirmed the diagnosis of severe pulmonary hypertension with right atrial pressure of 30 mmHg, right ventricular pressures of 112/20 mmHg, pulmonary artery pressures of 113/44 mmHg with a mean of 73. The pulmonary capillary wedge pressure was 28 mmHg with a transpulmonary gradient of 45 mmHg and pulmonary vascular resistance of 11.2 wood units. The ratio of pulmonary blood flow to systemic blood flow was 3.6 confirming the presence of left to right shunting. These results were consistent with mixed etiology of pulmonary hypertension with predominant pulmonary vascular disease [2].

Differential diagnosis

Other causes of pulmonary hypertension were ruled out by negative serology for hepatitis, autoimmune pathology, markers of inflammation and hypercoagulability. A ventilation-perfusion scan was negative for the thromboembolic disease. Pulmonary hypertension associated with Scimitar syndrome would be classified as Group 1 pulmonary hypertension associated with congenital cardiovascular malformations. The interstitial lung disease may have been due to her exposure to irritant fumes during her employment and would cause a component of Group 3 pulmonary hypertension. The patient had mixed etiology of pulmonary hypertension.

Treatment

Due to the absence of shunting, a small number of anomalous veins draining only a small part of the right lung, the absence of associated valvular abnormalities and the lack of any concomitant cardiac or pulmonary disease, medical management was chosen in our patient [3]. The patient was started on nifedipine and low dose aspirin. Later the patient developed paroxysmal atrial flutter. She was started on apixaban and her nifedipine was switched to diltiazem. The patient was referred to a pulmonary hypertension clinic at a tertiary center.

## Discussion

PAPVR is a rare congenital defect in which some of the pulmonary veins drain into the right side of the circulation. The right pulmonary veins are more often affected for unknown reasons [4]. There are four types of PAPVR that have been described as supracardiac, infracardiac, cardiac or mixed [5]. Infracardiac PAPVR involves most of the right lung draining into the inferior vena cava just above or below the right hemidiaphragm via the anomalous vein which appears as a Turkish curved sword on a chest X-ray (the eponymous Scimitar) [6]. This syndrome as originally described by Cooper in 1836 involved other coexisting cardiovascular and pulmonary malformations [7]. This is also called as the infantile variant of Scimitar syndrome as the patients present with significant symptoms during infancy. Presence of the Scimitar vein without any other malformation is called as incomplete Scimitar syndrome or adult variant of Scimitar syndrome with patients presenting after one year of age [8, 9].

Although patients with the incomplete Scimitar syndrome develop symptoms later due to small volume shunting of blood, it is rare to present in the seventies. Surgical repair is necessary in infantile Scimitar syndrome and is often undertaken in most patients presenting in childhood with incomplete Scimitar syndrome. A surgical strategy was not pursued in this case considering the low likelihood of benefit considering she had already developed severe pulmonary hypertension and did not have other factors that would indicate a potential for benefit from surgical repair (as mentioned in the Treatment section).

## Conclusions

Incomplete Scimitar syndrome is a form of infracardiac anomalous pulmonary venous return with the right lung draining into the inferior vena cava without any other cardiovascular malformations. It is a rare congenital anomaly that may present in late adulthood with dyspnea on exertion due to development of pulmonary hypertension. Medical management of pulmonary hypertension is the mainstay of therapy. Surgical interventions may not be necessary due to the low volume of the blood being shunted.

## References

[1] Huang SF, Yu WC, Chern JH (2011). Scimitar syndrome in an older adult. J Chin Med Assoc.

[2] Naeije R, Vachiery JL, Yerly P (2013). The transpulmonary pressure gradient for the diagnosis of pulmonary vascular disease. Eur Respir J.

[3] Sears EH, Aliotta JM, Klinger JR (2012). Partial anomalous pulmonary venous return presenting with adult-onset pulmonary hypertension. Pulm Circ.

[4] Koester S, Lee JZ, Lee KS (2016). Pulmonary hypertension secondary to partial anomalous pulmonary venous return in an elderly. Case Rep Cardiol.

[5] Aluja Jaramillo F, Hernandez C, Garzón JP (2017). Infracardiac type total anomalous pulmonary venous return with obstruction and dilatation of portal vein. Radiol Case Rep.

[6] Lee M (2007). Isolated and complex scimitar vein anomalies and their differentiation from the meandering right pulmonary vein. Yonsei Med J.

[7] Cooper G (1836). Case of malformation of the thoracic viscera consisting of imperfect development of the right lung and transposition of the heart. London Med Gaz.

[8] Sehgal A, Loughran-Fowlds A (2005). Scimitar syndrome. Indian J Pediatr.

[9] Trell E, Johansson BW, Andrén L (1971). The scimitar syndrome with particular reference to its pathogenesis. Z Kreislaufforsch.

